# Efficacy of Intraoperative Vein Graft Storage Solutions in Preserving Endothelial Cell Integrity during Coronary Artery Bypass Surgery

**DOI:** 10.3390/jcm11041093

**Published:** 2022-02-18

**Authors:** Francesca Toto, Tiziano Torre, Lucia Turchetto, Viviana Lo Cicero, Sabrina Soncin, Catherine Klersy, Stefanos Demertzis, Enrico Ferrari

**Affiliations:** 1 Cardiac Surgery Unit, Cardiocentro Ticino Institute, 6900 Lugano, Switzerland; francesca.toto@eoc.ch (F.T.); tiziano.torre@eoc.ch (T.T.); stefanos.demertzis@eoc.ch (S.D.); 2 Lugano Cell Factory, Cardiocentro Ticino Institute, 6900 Lugano, Switzerland; lucia.turchetto@eoc.ch (L.T.); viviana.locicero@eoc.ch (V.L.C.); sabrina.soncin@eoc.ch (S.S.); 3 Service of Clinical Epidemiology & Biometry, IRCCS Fondazione Policlinico San Matteo, 27100 Pavia, Italy; c.klersy@smatteo.pv.it; 4 Biomedicine Faculty, University of Italian Switzerland, 6900 Lugano, Switzerland

**Keywords:** coronary artery bypass grafting, saphenous vein graft, vein graft disease, vein graft failure

## Abstract

(1) Introduction: Intraoperative preservation solutions for saphenous vein grafts may influence the endothelial structure and increase the risk of graft failure after coronary surgery. The aim of the study was to compare the efficacy of three solutions in maintaining the endothelial cell integrity of venous segments. (2) Methods: We tested the efficacy of physiological saline solution (PSS), heparinized autologous blood (HAB) and DuraGraft^®^ in preserving the endothelium of vein segments by evaluating the degree of endothelial cell apoptosis. Two incubation times (2 and 4 h from harvesting) were used for each solution. The quantification of apoptotic cells was computed as the intensity nuclei/intensity area ratio. (3) Results: After 2 h of ischemia, the degree of apoptosis decreased progressively across the use of DuraGraft, HAB and PSS (*p* = 0.004), although only the difference between DuraGraft and PSS yielded a statistical significance (*p* = 0.002). After 4 h, a similar decrease in apoptosis was shown across the three media; however, statistical significance was not reached. The analysis of the elapsed time (2 or 4 h of incubation) showed that this was a relevant factor in maintaining the endothelial structural integrity independently from the storage solution (test for interaction of media and time *p* = 0.010). (4) Conclusion: Within 2 h of incubation, endothelial structural integrity depended on the incubating medium. DuraGraft better protected the SVG against ischemic-induced apoptosis when compared to saline solution. Prolonged ischemia was associated with extended endothelium damage and none of the studied solutions protected the vein graft.

## 1. Introduction

The saphenous vein graft (SVG) remains the most widely used conduit for non-left anterior descending coronary territories in patients undergoing coronary artery bypass grafting (CABG) [1,2]. Compared to the proven good long-term patency of arterial conduits, the SVG has a higher risk of developing vein graft disease, with subsequent vein graft failure and increased risk of adverse cardiac events [3,4,5]. Pathological studies have demonstrated that the endothelial damage occurring during vein harvesting and manipulation was an important cause of failure but ex vivo studies have also highlighted the importance of the storage solution in which the vein is temporarily immersed before implantation [6,7]. These solutions can preserve the endothelium integrity while reducing ischaemic reperfusion injury occurring between the harvesting and the completion of the anastomoses with blood reperfusion [8,9]. To date, physiological saline solution (PSS) and heparinized autologous blood (HAB) are the most commonly used solutions for graft preservation in CABG [5,8,9,10,11]. However, in a sub-analysis of the PREVENT IV trial it was demonstrated that neither PSS nor HAB were effective in protecting the SVG during the storage and endothelium damage was correlated with a higher degree of vein graft failure after 1 year [11]. There is a need to test alternative solutions that can improve long-term SVG patency. DuraGraft^®^ (Somahlution, Jupiter, FL, USA) is a specifically designed tissue preservation solution containing powerful antioxidant and cellular reducing agents that was developed to prevent ischaemic reperfusion injury and protect structural and functional vein integrity. In this study, we evaluated the endothelial cell integrity of fresh ex vivo SVG segments stored in three different solutions: saline solution, heparinized autologous blood and DuraGraft.

## 2. Methods

### 2.1. Saphenous Vein Segments and Experimental Conditions

Saphenous vein segments were harvested from 12 male patients undergoing coronary surgery at our institution and patients provided their written informed consent before surgery. Patients also signed an informed consent for the use of their anonymized clinical data and biological specimens for non-genetic research purposes. The present investigation abides by the principles outlined in the Declaration of Helsinki (Ethical Principles for Medical Research Involving Human Subjects) adopted by the 18th WMA General Assembly in Helsinki, Finland.

Patients presented with typical cardiovascular risk profiles, including diabetes, hypertension and dyslipidaemia. Pre-operatively, patients were under treatment with standard medications for coronary disease such as aspirin, beta-blockers and lipid-lowering agents (e.g., statins). Routinely, all patients underwent a preoperative evaluation of SVG diameters using ultrasound assessment as well as a clinical evaluation by the surgeon. Those SVGs that showed varices or a luminal diameter less than 2 mm were not included in the present study. The SVG was harvested from the distal leg in all patients using the skin bridge method and optimal handling techniques (e.g., avoidance of over pressurization during checking for leakage, excessive handling and distortion) in order to reduce traumatic damages to the conduit’s endothelium. For the purpose of this study, the first 3 cm of the SVG were harvested and immediately removed for the analysis, in order to minimize the trauma.

**Figure 1:**
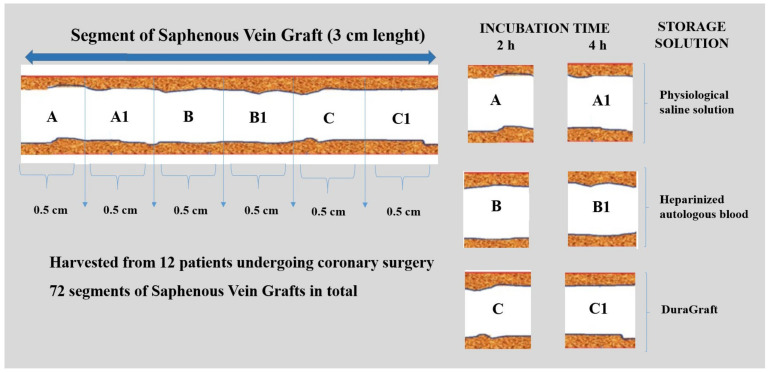
Experimental condition. Each segment of fresh saphenous vein was divided into six sections that were 0.5 cm in length. Two incubation time sets were tested: 2 h and 4 h for each vein preservation solution.

Each saphenous segment was divided into six sections that were 0.5 cm in length and then each section was separately stored in a Falcon Tube (50 mL) with 6 mL of storage solution (total: 72 segments) (Figure 1).

The selection of the sample size length of 0.5 cm was based on the principle of having enough vein length to perform the study with meaningful results and not to waste biological human material.

Three different solutions for saphenous graft storage were tested: physiological saline solution, heparinized autologous blood (heparin: 40 U/mL) and DuraGraft. Vein segments were carefully flushed and stored in one of the preserving solutions as indicated and then incubated at 22 °C (24 segments each). Two incubation time sets were fixed for each vein preservation solution: 2 h and 4 h from the surgical harvesting. Measurements were made after 2 h of ischemia, which represented the longest SVG ischemic time that could happen when coronary surgery is performed in combination with complex or multiple valve surgery or aortic surgery. This was a limit that, nowadays, is achieved less rarely than in the past given the number of more complex cases that cardiac surgeons are faced with. The 4 h time set reproduced the longest intraoperative lapse of time (therefore ischemia) that could elapse between the vein harvesting and the vein implantation during very complex and rare cardiac surgery cases and that could be used as a control group.

After 2 h or 4 h of incubation, vein segments were fixed in 4% paraformaldehyde and then embedded in solid OCT blocks using liquid nitrogen. Once the complete solidification at −20 °C was achieved, the blocks were cut with cryostat in order to obtain six slides for each saphenous vein segment, with five sections/slide.

### 2.2. The Assay Principle

The DeadEnd™ Fluorometric TUNEL System (Promega G3250) (Promega Corporation, Madison, WI, USA) was used for specific detection and quantification of apoptotic cells by measuring the nuclear DNA fragmentation, an important biochemical hallmark of apoptosis in many cell types. The DeadEnd™ Fluorometric TUNEL System quantified the fragmented DNA of the apoptotic cells by catalytically incorporating fluorescein-12-dUTP at 3′-OH DNA ends using the Terminal Deoxynucleotidyl Transferase, Recombinant, enzyme (rTdT). rTdT, which formed a polymeric tail using the principle of the TUNEL (TdT-mediated dUTP Nick-End Labeling) assay. Fluorescence microscopy was used to directly visualize the fluorescein-12-dUTP labelled DNA.

### 2.3. Assay Protocol

**Figure 2:**
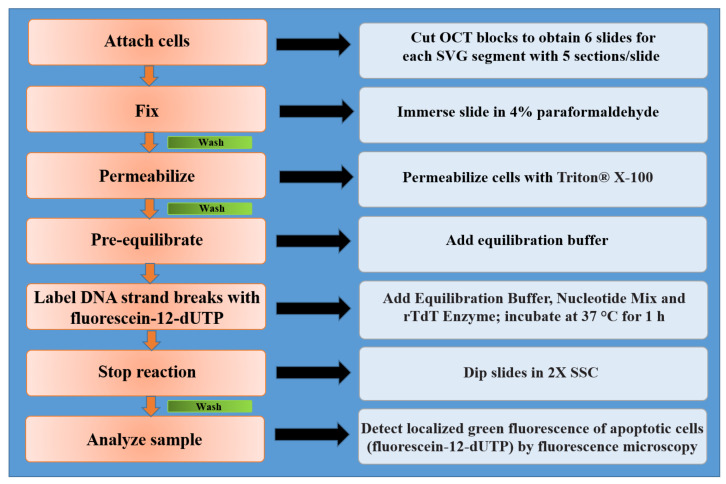
Protocol overview for use of the DeadEnd™ Fluorometric TUNEL System in fluorescence microscopy of the attached cells.

After the fixing phase, slides were immersed in 0.5% Triton^®^ X-100 (Merck KGaA, Darmstadt, Germany) solution for the permeabilization step. An optional positive control slide using the DNase I treatment was prepared. The DNase I treatment of the fixed cells resulted in fragmentation of the chromosomal DNA and exposure of multiple 3′-OH DNA ends for labelling. The next phase for samples and positive control was the equilibration phase, which was started by covering the cells with 100 μL of equilibration buffer (200 mM potassium cacodylate pH 6.6 at 25 °C; 25 mM Tris-HCl pH 6.6 at 25 °C; 0.2 mM DTT; 0.25 mg/mL BSA; 2.5 mM cobalt chloride). While tissues were equilibrating, the nucleotide mix that was on ice was thawed and sufficient rTdT incubation buffer was prepared for all experimental and positive control reactions. The incubation buffer contained equilibration buffer, the nucleotide mix (50 μM fluorescein-12-dUTP; 100 μM dATP; 10 mM Tris-HCl pH 7.6; 1 mM EDTA) and rTdT enzymes. The rTdT incubation buffer was added to the sample slides and positive control, which were covered with a plastic coverslip and incubated in a humidified chamber at 37 °C for 1 h. At the same time, a negative control was created by adding equilibration buffer and the nucleotide mix. During this phase, the fragmented DNA of the apoptotic cells was measured by catalytically incorporating fluorescein-12-dUTP at the 3′-OH DNA ends using the rTdT enzyme. Avoiding exposure to light was imperative; in order to stop the tailing reaction the sample slides and negative and positive controls were immersed in 2X SSC solution 20X SCC (87.7 g NaCl; 44.1 g sodium citrate). Finally, the samples were immediately analyzed under a fluorescence microscope using a standard fluorescein filter set to view the green fluorescence at 520 ± 20 nm. Fluorescein-12-dUTP incorporation resulted in localized high green fluorescence within the nucleus of the apoptotic cells. All processes are summarized in Figure 2.

### 2.4. Detection and Quantification of Apoptotic Cells

To detect and quantify apoptotic cells we used the software “ImageJ” (NIH Image, Bethesda, MD, USA), a freely available java-based, public-domain image processing and analysis program developed at the National Institutes of Health. The quantification of apoptotic cells was obtained by calculating the ratio between apoptotic cell fluorescence intensity (intensity nuclei) over the vein slide total area intensity (intensity area) expressed as a percentage (intensity nuclei/intensity area ratio).

### 2.5. Statistical Analysis

We used Stata 16 (StataCorp, College Station, TX, USA) to perform computations. We considered a 2-sided *p*-value < 0.05 as significant. We used the Bonferroni correction for post-hoc comparisons and set the *p*-value at 0.017 for significance. We fitted generalized linear regression models to assess the role of treatment on the intensity nuclei/intensity area ratio (%) at time 2 h and 4 h, separately. We calculated robust Huber–White standard errors to account for the intra-patient correlation of measures. We tested the interaction of treatments and time to verify whether the effect of treatment depended on time. This being the case, we resent 2 separate models for the 2 and 4 h incubation times. Given its distribution, we applied a normalizing natural logarithmic transformation to the intensity nuclei/intensity area ratio (%) before its inclusion in the model. We derived differences (on the log scale) between treatment groups and 95% confidence intervals from the model. For descriptive purposes, we averaged measures over each patient within treatment and time. We then computed the mean and standard deviation (SD) by treatment arm and time, while weighting for the number of measures used for the patient average (stata analytic weights were used).

## 3. Results

### 3.1. Effects of Preservation Solutions on Vein Graft Segments at 2 h Incubation Time

**Figure 3:**
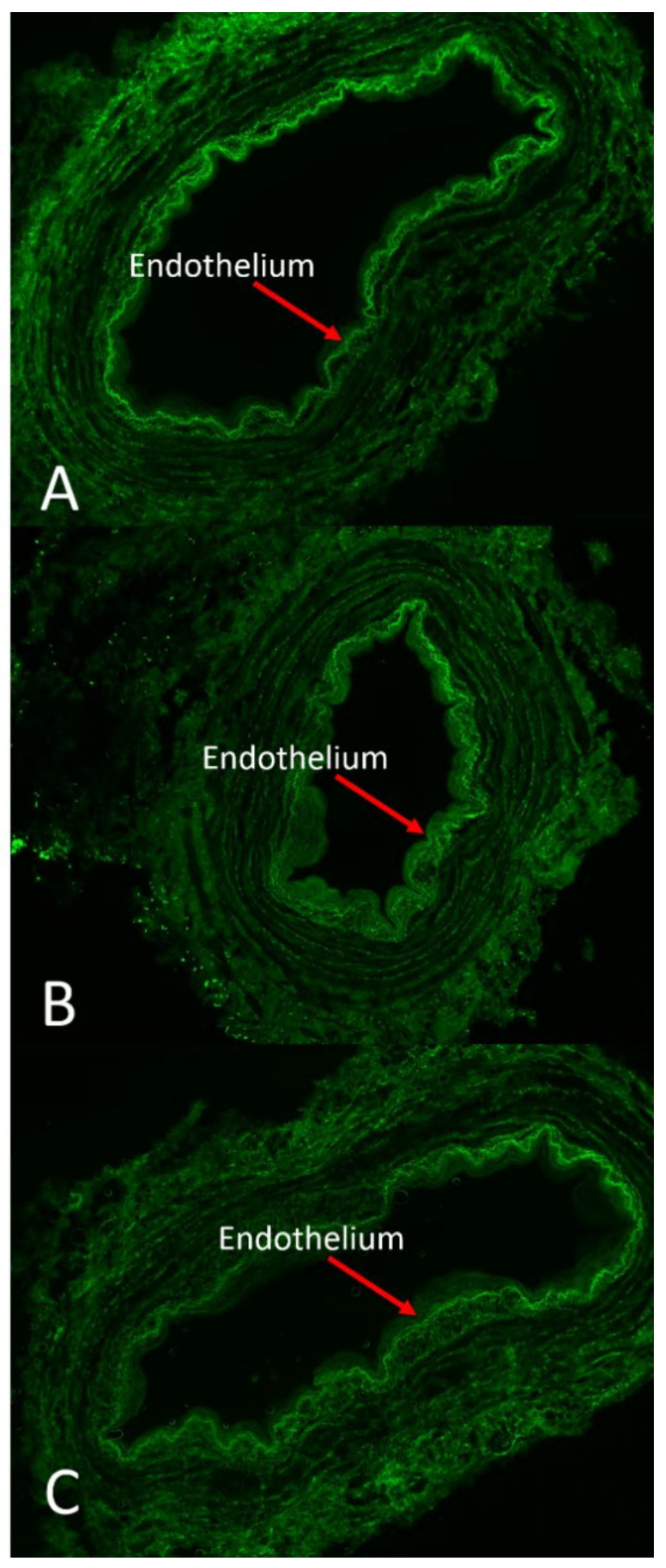
Changes in endothelial viability of vein segments stored in different preservation solutions. Vein segments were stored in DuraGraft (**A**), autologous heparinized blood (AHB) (**B**) and physiological salt solution (PSS) (**C**) for 2 h after vein harvesting. The high green fluorescence indicates cell apoptosis. Extensive endothelial cell death was observed in vessels preserved in PSS. Cell viability was moderately preserved in AHB. Endothelial cells remained viable in vessels preserved in DuraGraft solution throughout the 2 h of storage. The test used measured the fragmented DNA of the apoptotic cells by catalytically incorporating fluorescein-12-dUTP(a) at the 3′-OH DNA ends. For these reason the test did not discern between living and dead cells. Magnification 4X.

The effect of DuraGraft solution on endothelial structural integrity was compared to HAB and PSS (Figure 3).

**Figure 4:**
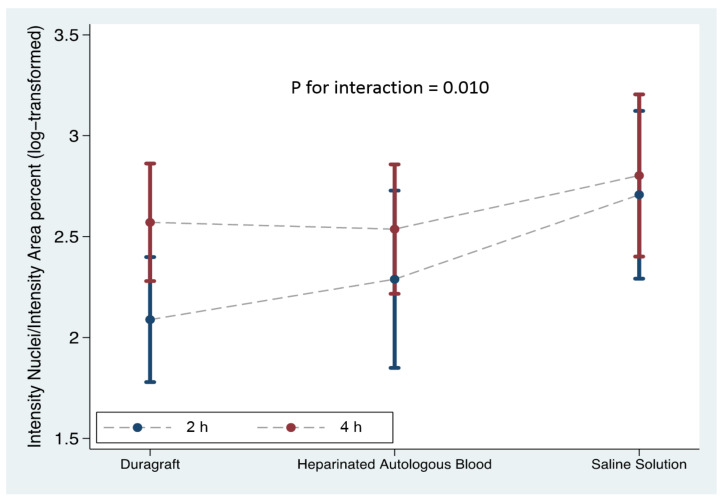
The comparison between the three different storage solutions at the two time sets: the blue line represents the preservation of the vein graft at 2 h from vein harvesting; the red line indicates the preservation of the vein graft at 4 h from vein harvesting. Intensity nuclei/intensity area (percent) was log transformed for model fitting.

Vein segments stored in physiological saline exhibited a robust green fluorescence pattern in the luminal region indicative of extensive cell damage and compromised viability of endothelial cells; a fair amount of dead cells (green fluorescence) was observed in vein segments stored in HAB, while well-preserved endothelium remained structurally intact without green fluorescence dots inside the cells in vein segments stored in DuraGraft. Our analysis showed that the role of preservation solution on the intensity nuclei/intensity area ratio after 2 h of vein storage from harvesting was relevant (Table 1), and there was an increase in the intensity nuclei/intensity area ratio over DuraGraft, HAB and PSS (Figure 4; model *p* = 0.004).

Although DuraGraft preservation was more effective in protecting the vein grafts (intensity nuclei/intensity area ratio of 10.11 ± 5.81% for Duragraft vs. 13.12 ± 7.10% for HAB) no statistical significance was reached (*p* = 0.193). Conversely, the intensity nuclei/intensity area ratio was significantly lower (*p* = 0.002) for DuraGraft versus PSS (10.11 ± 5.81% for Duragraft vs. 19.44 ± 10.68%, respectively).

### 3.2. Effects of Preservation Solutions on Vein Graft Segments at 4 h Incubation Time

**Figure 5:**
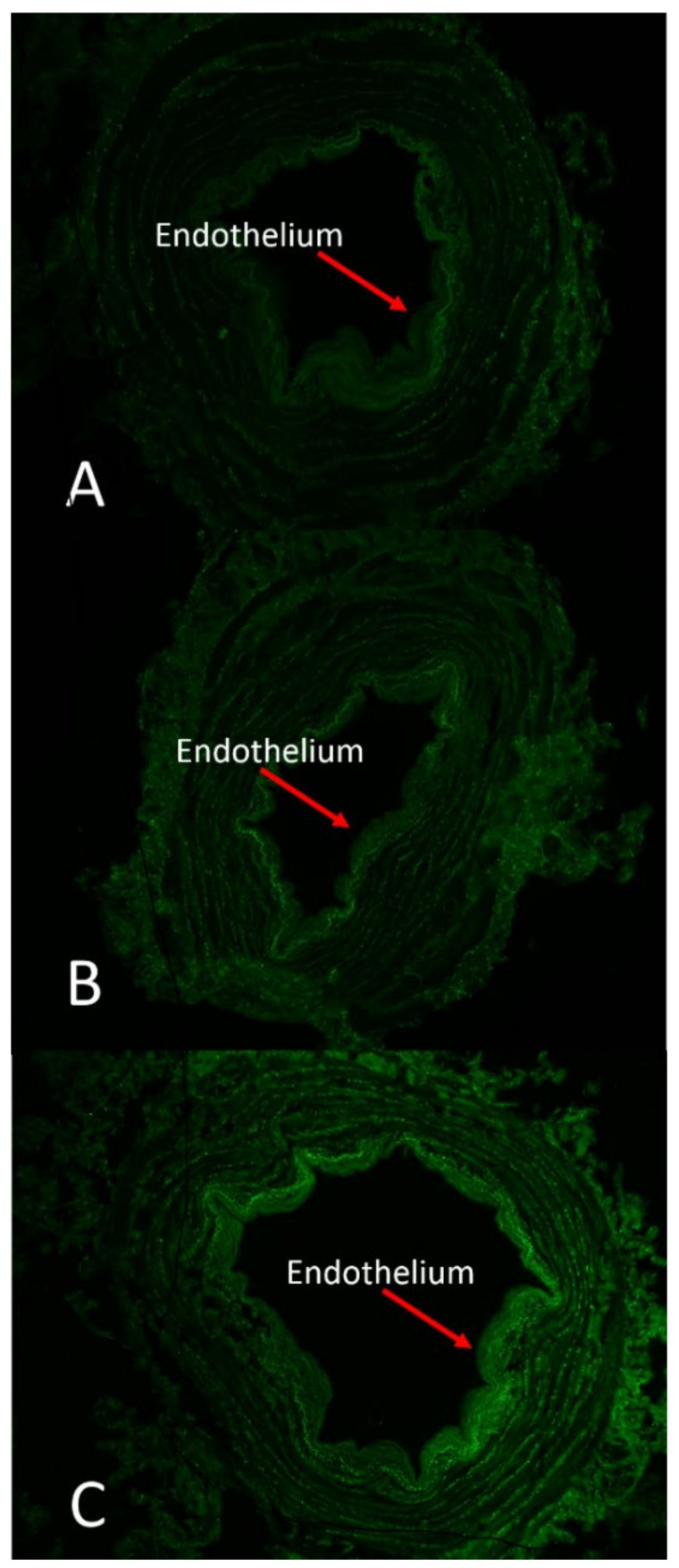
Changes in endothelial viability of vein segments stored in different preservation solutions. Vein segments were stored in Duragraft (**A**), autologous heparinized blood (AHB) (**B**) and physiological salt solution (PSS) (**C**) for 4 h after vein harvesting. The high green fluorescence indicates the apoptotic cells. At this incubation time, no differences were observed between the three intraoperative storage solutions regarding endothelial cell death. The test used measured the fragmented DNA of the apoptotic cells by catalytically incorporating fluorescein-12-dUTP(a) at the 3′-OH DNA ends. For this reason the test dis not discern between living and dead cells. Magnification 4X.

The comparison of DuraGraft solution, HAB and PSS effects on endothelial structural integrity after 4 h of storage are showed in Table 1 and Figure 5.

The endothelial monolayer was juxtaposed with the lumen, which showed a significant amount of loss of cellular integrity during extensive storage in PSS and HAB. A lesser degree of green fluorescence, indicative of cell apoptosis, was observed in vein segments preserved in DuraGraft storage. However, none of the examined intraoperative solutions proved to be more effective in maintaining the structural integrity of the endothelial layer after four hours from vein harvesting (model *p* = 0.168). We observed a modest increase in the mean values of the intensity nuclei/intensity area ratio over the DuraGraft, HAB and PSS solutions, from 15% to 16% and 21%, as compared to the 2 h incubation data (Table 1 and Figure 4).

### 3.3. Interaction of Treatment and Time

As endothelial damage appears to be a major cause of graft failure, defining the time of this injury is of prime importance. We evaluated the interaction between treatment and time. Time from vein graft harvesting has been a relevant factor in maintaining the structural integrity of vein grafts independently from the type of storage solution (test for interaction *p* = 0.010) (Table 1). After 4 h from vein harvesting, experimental conditions showed extensive endothelial disruption in the endothelial monolayer and none of the examined storage solutions were effective in preserving vein integrity.

## 4. Discussion

Keeping the structure and function of the saphenous vein endothelial layer intact during ischaemic storage is a crucial step in preventing venous failure after CABG. Vein graft disease represents the clinical manifestation of an ischemic reperfusion injury initiated during intraoperative ischemic episodes via oxidative damage and metabolic stress, which can be perpetuated by post-reperfusion responses to the damaged endothelium [12,13,14,15]. The effective protection of SVG endothelium during surgery is of crucial importance and the use of an appropriate preservation solution during vein storage should mitigate the ischemic reperfusion injury and prevent damage. As a matter of fact, the suboptimal preservation of the SVG is associated with endothelial damages in ex vivo studies and the PREVENT IV trail confirmed the higher rate of one-year graft failure for veins stored in saline or blood-based solutions when compared with a buffered solution [8,9,11].

In the present study, we were able to assess and compare the efficacy of saline solution, heparinised blood and DuraGraft in preserving the endothelial structure of saphenous vein grafts by evaluating the percentage of endothelial cell apoptosis in an ex vivo experiment after 2 and 4 h of ischemia from surgical vein harvesting. The main results are the following:

1. After two hours of ischemia, DuraGraft was the most effective storage solution and was associated with a lower rate of cell apoptosis, followed by HAB and, lastly, PSS.
2. After four hours of ischemia, none of the examined treatments were efficient in protecting the vein graft endothelium against structural decay.

With regards to the 2 h ischemic period, the most significant difference was established when comparing DuraGraft and the physiological saline solution. This standard preservation did not represent a good storage solution for supporting endothelial or smooth muscle cells, lacked an energy source, such as glucose, and exerted potential “solution damage” given its lack of ionic balance and its acidic pH (5.5), which is dangerous for the fragile endothelial cells. Given the observation of a progressive increase in the intensity nuclei/intensity area ratio across the three solutions, we tentatively suggest DuraGraft to be the most valid preservation solution up to two hours from vein graft harvesting. The lack of statistical significance when compared to HAB, however, prompts the need for further, larger, studies to confirm this encouraging finding. The same intraoperative storage solutions were compared at four hours from saphenous segment explant. During prolonged ex vivo storage of harvested saphenous veins, an increase in anaerobic metabolism was observed. As a result, lactic acid accumulated with significant increase in acidosis and a concomitant decrease in pH. Hence, this interval of time may have affected both the structure and the function of the vein graft, depending on the composition and the temperature of the storage solution. We could conclude that after four hours from vein graft harvesting, the endothelial damage was too extensive and none of the available preservation solutions could protect the endothelium layer from ischaemic damage.

A key factor may be the temperature of the storage solutions, as a low temperature is able to slow metabolic processes and consequently cell death. In our study, vein grafts were stored at room temperature (the same temperature as an operating room). This storage condition may not have provided an efficient environment that was capable of preserving endothelial structures from tissue ischaemiac injury during the 4 h incubation time. However, a storage time of two hours reproduced the longest intraoperative time that usually elapses from the vein harvesting to the coronary (and bypass graft) reperfusion, especially when complex or combined cardiac procedures are concerned. The 4 h time represented a control group and the longest possible vein storage time in cardiac surgery (rare condition).

These results were in line with other published studies showing that vessel storage in PSS, HAB and other solutions led to endothelium damage, perhaps because of free radical injury, low pH, storage media composition or hostile environments [16,17,18,19,20]. Thatte and colleagues, demonstrated that human saphenous vein segments stored in DuraGraft (original name GALA: glutathione, ascorbic acid, L-arginine) showed enhanced endothelial structural integrity preservation under light microscopy compared to a range of solutions, including saline, HAB and Hank’s balanced salt solution. Moreover, the multiphoton imaging of the endothelium confirmed a high level of structural viability and few dead cells in DuraGraft-treated grafts following 24 h of cold ischemia versus >90% loss of cell viability and functionality in grafts preserved in blood, saline or buffered salt solution [8].

Pachuk et al. reported molecular damage in pig mammary veins following 45 min of exposure to saline, displaying a lower expression of endothelial cell surface proteins in immunohistochemistry staining compared with veins stored in DuraGraft [21]. In the same analysis, a significant loss of human saphenous vein graft-cell viability had already occurred after 15 min of exposure to saline solution, with an almost complete loss occurring within 30 min. whereas cell viability was maintained during up to 5 h of exposure to cold DuraGraft solution. Moreover, cytotoxicity assays demonstrated that saline-induced microscopically visible cell damage occurred within 60 min, while DuraGraft-treated cells did not show evidence of damage or reactivity.

Clinically, a US observational study showed that in patients undergoing CABG SVG treatment with DuraGraft was associated with a lower rate of long-term adverse events, including myocardial infarction and need for repeat revascularization, which suggested the potential benefit of the intraoperative use of DuraGraft in reducing graft disease-related adverse events [22]. To further assess these promising clinical results, a prospective, double-blind, randomized trial comparing DuraGraft-treated SVG with saline-treated SVG in the same patient undergoing isolated CABG, has been recently designed: the aim of the study was to evaluate the graft remodeling behavior after Duragraft treatment by using multidetector computed tomography to evaluate early anatomical markers of graft disease [23]. Additionally, a European multi-centre DuraGraft Registry has been created in order to assess the long-term outcome and quality of life of patients undergoing CABG with venous grafts treated with DuraGraft [24].

The results of the present study could have a consistent clinical impact considering that, despite extensive evidences of their inefficient protection of SVG endothelium, saline solution and autologous blood continue to be employed in several centers for intraoperative graft preservation worldwide. This was emphasized in a recent survey involving 100 top-performing US hospitals, which reported that saline solution was used in 26 centers (28.9%) and autologous blood in was used in 24 centers (26.7%) [10]. From this perspective, the composition of DuraGraft may represent a valid protective solution for short-term graft storage. It is formulated into an ionically and pH-balanced physiological salt solution containing antioxidants to mitigate oxidative damage, glucose, arginine and high-energy phosphates to reduce metabolic stress during ischemia, providing a favorable environment and cellular support during ex vivo SVG storage.

### Limits of the Study

This study had some limitations. First, a relatively small sample size was used; further studies are needed to validate our findings. Second, this was a single-center study, so that only the surgical practice performed at our institution was captured. Of note is the fact that the team members and the method of vein harvesting, handling and exposure to storage solution did not vary over the study period. Multi-center studies are warranted for confirmation. Third, our cohort of patients was exclusively composed of male and primarily Caucasian white patients; thus, our results may not be generalizable to women, and other racial groups.

## 5. Conclusions

Our study suggested that DuraGraft storage solution provided more effective vein endothelium protection against disruption of flow induced apoptosis when compared to the most common storage solutions and during a period of time of 2 h from surgical harvesting. A longer time of ischemia may be associated with extended endothelium damage and none of the tested solutions were effective in protecting the vein graft.

## Figures and Tables

**Table 1:** Differences between treatment arms at 2 and 4 h (generalized linear regression model). Note: Intensity nuclei/intensity area (percent) was log transformed * for model fitting. Given that the interaction of time and type of solution was present, we presented separate models after 2 and 4 h of incubation.

	2 h of Incubation			4 h of Incubation			Interaction of Treatment and Time
Storage Solution	Mean (SD)	Regression Model * Difference vs. DuraGraft (95%CI)	*p*-Value	Mean (SD)	Regression Model * Difference vs. DuraGraft (95%CI)	*p*-Value	*p*-Value
	***Intensity Nuclei/Intensity Area (%)***		**0.004**	***Intensity Nuclei/Intensity Area (%)***		**0.168**	**0.010**
**DuraGraft**	10.11 ± 5.81	0		14.98 ± 5.58	0		
**Heparinized Autologous Blood**	13.12 ± 7.10	0.20 (−0.12–0.52)	0.193	16.01 ± 7.23	−0.03 (−0.28–0.21)	0.786	
**Saline Solution**	19.44 ± 10.68	0.62 (0.29–0.95) ^	0.002	20.83 ± 10.34	0.23 (−0.06–0.53)	0.110	

^ vs. heparinized autologous blood Δ 0.42 (95%CI −0.19 to 1.02), *p* = 0.077.

## Data Availability

The dataset generated and analysed during the current study is available from corresponding author upon reasonable request.

## Glossary of Abbreviations

SVG, saphenous vein graft; CABG, coronary artery bypass grafting; PSS, physiological saline solution; HAB, heparinized autologous blood; PBS, phosphate-buffered saline.
